# On the relationship between internet addiction and ADHD symptoms in adults: does the type of online activity matter?

**DOI:** 10.1186/s12889-025-23040-4

**Published:** 2025-06-04

**Authors:** Tuba Aydın, Benjamin A. Parris, Gizem Arabacı, Marina Kilintari, Jacqui Taylor

**Affiliations:** Department of Psychology, Faculty of Science and Technology, Bournemouth University, Bournemouth, UK

**Keywords:** ADHD, Inattention, Internet addiction, Inhibition, Online activities

## Abstract

**Background:**

Studies shows that there is a relationship between internet addiction and ADHD symptoms, especially inattention. A study found that there is a unique relation between each core ADHD symptom and different types of internet activities. Another study found that deficits in inhibitory control moderate the relationship between internet addiction and inattention. Therefore, this study aimed to explore how specific online activities might modify the relationship between inattention, internet addiction and inhibitory control.

**Methods:**

205 participants (79 females,126 males) between 18 and 49 years old took part in the study from a community sample. They completed the demographic information form, Adult ADHD Self-Report Scale Symptom (ASRS-v1.1) Checklist, and Young’s Internet Addiction Test (IAT) online. Then, they performed the Stroop Task.

**Results:**

Consistent with previous research we show that inattention predicts internet addiction, and that this relationship is moderated by inhibitory control ability; the relationship between inattention and internet addiction strengthens as executive function impairment increases.

**Conclusions:**

These results indicate that ADHD symptoms and executive function impairments interact in a way that leads to real-life consequences, even when there is no relationship between those symptoms and executive function performance. Furthermore, we show that no single online activity is responsible for the relationship between inattention and internet addiction, nor does inhibitory control ability determine which online activity participants engage in.

## Background

In the mid-1990s, the term “Internet addiction” (IA) was first used in the medical literature [1, 2]. It refers to a behavioural pattern of internet use that includes a dysfunctional craving to use the internet for uncontrolled and excessive amounts of time, along with significant psychosocial and functional impairments that cannot be explained by any other disorder [1]. However, the definition and conceptualising of internet addiction is a controversial issue. For example, due to there being insufficient peer-reviewed research to determine the diagnostic standards required to classify internet addiction as a mental disorder, it is not included in Diagnostic and Statistical Manual of Mental Disorders, Fifth Edition [3]. Despite issues in conceptualising IA, its prevalence is rising leading to healthcare costs; a meta-analysis in 2020 reported a prevalence of 7.02% (95% CI, 6.09–8.08%) [4], while the healthcare costs of Korean adolescents for internet addiction were estimated at over 29 billion dollars [5]. Therefore, it continues to be an emerging issue and as such a better understanding of IA is a social and financial imperative.

One question in internet addiction research is whether it is an addiction to a single entity or an addiction to more than one internet related activity (e.g., social networking, gaming, gambling, accessing online sources, and entertainment) [6, 7]. Some studies conclude that internet users choose to consciously participate in particular activities with particular content [8, 9], indicating the existence of specific internet use-related addictions, rather than a generalized internet addiction [10].

Internet addiction, whether it is a generalized internet addiction or is a specific internet use-related addiction, has comorbidities with many psychological disorders such as depressive disorders [11], autism spectrum disorders [12], anxiety [13], alcohol abuse [14], and Attention Deficit Hyperactivity Disorder (ADHD) [15]. ADHD is generally defined as a childhood neurodevelopmental disorder [16], however, research has shown that ADHD can occur in adulthood too [17]. This has led researchers to investigate whether adult ADHD is a continuation of childhood ADHD or adult-onset ADHD. A recent global systematic review and meta-analysis concluded that whereas the prevalence of adult ADHD with childhood onset was 2.58%, the prevalence of adult ADHD regardless of childhood onset was 6.76%. This means that 139.84 million and 366.33 million adults were affected by ADHD in 2020 globally [16]. For adult-onset ADHD, it is proposed that the adults can have had subthreshold signs of the disease when they were younger but meet diagnostic criteria in adulthood [18].

Inattention, hyperactivity, and impulsivity are traits found throughout the general population [19]; and an individual is diagnosed with clinical ADHD only when these symptoms are present at an extreme level. Trait level symptomatology is supported by studies showing that people with ADHD symptoms below the diagnostic threshold levels, share common features in brain activity [20] and genetics [21] with clinically diagnosed ADHD. However, as the severity of the disease increases, the deficits in different domains increase such as in behaviour problems, cognitive performance, family life [22], and executive functions (EFs) [23]. Whilst most research on ADHD without consideration of the individual core symptoms (most research is conducted on those with ADHD of the combined subtype with little consideration given to the predominantly inattention or hyperactive-impulsive subtypes), particular impairments have been theoretically linked to the different core symptoms [24, 25]. Recent work has linked specific cognitive and behavioural deficits such as mind wandering [26] and task-switching impairments [27] to inattention.

EFs can be defined as a group of top-down cognitive processes such as attention, reasoning, problem solving [28], emotion regulation, self-regulation [29], inhibition, working memory, and shifting [30]. In psychological research, inhibition, working memory, and shifting are the most frequently assessed executive functions and are considered as the core executive functions [31]. Deficits in EFs are a key component in many different clinical conditions [29] including in ADHD [23] and internet addiction [32]. Consistent with the notion that inattention and hyperactive-impulsive symptoms might have different cognitive and behaviour implications, Diamond (2013) theorised that inattention was linked to a working memory deficit [25], whilst Barkley (1997) theorised that inhibition deficits might result in hyperactive/impulsive symptoms [24].

In examining current literature, it can be seen that there is a link between ADHD and internet addiction [15, 33]. For example, a systematic review and meta-analysis concluded that individuals with internet addiction have more severe symptoms of ADHD [34]. Some research focused on the relationship at the symptom level of ADHD (e.g., inattention, hyperactivity, and impulsivity), and found differing results. For instance, while some studies reported that both inattention and hyperactivity were associated with internet addiction [33, 35, 36], another study found that only inattention symptoms were associated with internet addiction [37]. Furthermore, some studies have concluded that inattention is a unique predictor of internet addiction [34, 35, 37]. In all of these studies, internet addiction was conceptualised as a generalized internet addiction. In the present study we aimed to investigate whether the relationship between internet addiction and ADHD symptoms is the result of a specific type of online activity.

Zhang et al., (2022) showed that there is a unique relation between each core ADHD symptom and different types of Internet activities [38]. While only hyperactivity/impulsivity was related with online shopping, both inattention and hyperactivity/impulsivity were negatively correlated with information downloading and online learning, and positively correlated with online gaming [34]. Another study that investigated ADHD symptoms, internet addiction, and online activities concluded that there is no group difference in online activities between low and high ADHD groups, and also between problematic internet usage and non-internet addiction groups [39]. Furthermore, they reported that while the most commonly reported online activities in the problematic internet use group were video surfing, video game watching, playing MMORPGs, and watching television, for people in the high ADHD group, the most commonly reported online activities were online gaming, video game watching, MMORPGs, online gambling, and television [35].

In this study, we aimed to further investigate the relationship between inattention and internet addiction from an online activities’ perspective following Panagiotidi & Overton (2018) [39]. Whilst the focus of much of our work has been on the cognitive and behavioural implications of inattention [26, 27, 35, 37, 40], we also consider and analyse the symptoms hyperactivity and impulsivity in their relation to the topic of interest because it permits us to investigate what is uniquely a result of or mainly the result of inattention. Therefore, it was planned to use T-test and Chi-square for multiple response sets. A further aim of the study was to replicate our previous finding showing inhibitory control ability moderated the relationship between internet addiction and inattention [37], and to see if inhibitory control ability is related to the type of online activity participants engage in. Therefore, it was planned to use mixed ANOVA, T-tests, a stepwise regression analysis, and moderation analysis following our previous research.

## Methods

### Participants

Two hundred and twenty-nine participants were included in the study with the inclusion criteria: (1) being above 18 years old; (2) being fluent in English, (3) currently not receiving psychological or neurological treatment, and (4) normal or corrected-to-normal vision. Two participants reported that they are undergoing psychological treatment and were excluded from the dataset. The normality of inattention symptom, hyperactivity/impulsivity, total ASRS scores, IAT, Stroop interference score and Stroop facilitation scores were checked with Kolmogorov- Smirnov test. It was found that inattention symptom, hyperactivity/impulsivity, total ASRS scores, and IAT were not normally distributed. Normality tests were performed until the inattention score and internet addiction score showed normal distribution according to the Boxplots. Following this procedure, as they were marked as outliers in the boxplots, twenty-two outliers were removed from the data set. 205 participants (79 females,126 males) between 18 and 49 years old remained (*M*_age_=30.62; *SD* = 7.40).

Based on inattention symptom scores and IA scores, participants were grouped as low (*n* = 112, *M* = 7.83, *SD* = 3.38) and high inattention groups (*n* = 93, *M* = 17.65, *SD* = 3.87), and low (*n* = 101, *M* = 19.08, *SD* = 6.95) and high (*n* = 104, *M* = 38.32, *SD* = 7.78) internet addiction groups based on their mean scores. Participants whose score was less than the mean score of inattention and internet addiction were classified as low inattention group or low internet addiction group, while participants whose score was more than the mean score of inattention and internet addiction classified as high inattention group or high internet addiction group. Based on inattention score, there was group differences between low and high inattention group *t*(203) = -19.37, *p* = < 0.001. Based on internet addiction score, there was group differences between low and high internet addiction group *t*(203) = -18.64, *p* = < 0.001. Based on gender, while there were no group differences between low and high internet addiction group (*p* =.260), there were group differences between low and high inattention group (*p* =.008). Based on age, while there were no group differences between low and high inattention group (*p* =.141), there were group differences between low and high internet addiction group (*p* = < 0.001).

Most participants were recruited from Prolific and received £6 compensation, whilst other participants were recruited via social media adverts with no compensation. The study was approved by the Ethics committee of Bournemouth University (ID: 55657). All participants gave informed consent. All procedures in the study were aligned with The Helsinki Declaration of 1975, as amended in 2013, and the ethical guidelines of the relevant committee on human testing (both national and institutional) [41].

### Design

The study had a 2 (inattention: high, low) x 2 (internet addiction: high, low) x 3 (Stroop conditions: congruent, incongruent, and neutral) x 9 (online activities) mixed design. With inattention and internet addiction groups as the independent variables and RTs on the Stroop task and top 5 online activities and duration participants engaged in online activities as the dependent variables.

### Procedure

Firstly, all participants accessed the Participant Information Sheet and Participant Agreement Form online. After consenting to participate in the study, they completed the demographic information form, Adult ADHD Self-Report Scale Symptom (ASRS-v1.1) Checklist, and Young’s Internet Addiction Test (IAT) online in this order. At the end of the IAT, a link directing participant to the Stroop Task was presented. Then, they performed the Stroop Task.

### The Stroop task

The Stroop Task was completed using Testable (www.testable.org) and consisted of three types of trials: congruent trials (e.g., the word “green” written in green ink), incongruent trials (e.g., the word “yellow” written in red ink) and neutral trials (e.g., the word “ship” written in blue ink). At the beginning, twelve practice trials of each trial type (36 in total) were given to each participant. After the practice session, participants performed 288 randomly presented trials (96 neutrals, 96 incongruent and 96 congruent trials). At the start of each trial, participants were presented with a black fixation cross in the centre of a grey screen for 500 milliseconds (ms). This was followed by the Stroop stimulus, presented at the centre of the screen in Aptos Narrow Body font, size 12, in all caps which remained at the centre of the screen until a response was executed. Participants were instructed to press the assigned key corresponding to the colour of the text as quickly and accurately as possible while ignoring the word’s meaning with a maximum response window of 2000ms. Participants pressed the” c” for blue, the “v” key for red, and the “b” key for yellow and the “n” key for green responses. Upon committing an error, an additional visual error message (‘X’) was presented in black (font size = Testable’s + 5) for 500 ms followed by a blank screen of 100 ms. The task was completed in ~ 16 min.”

### Questionnaires

#### Demographic information form

In the demographic information form, participants were asked to report their age, gender, dominant hand, vision, and receiving of psychological or neurological treatment. In addition, participants were asked to report their five online activities they engage in most often and usage time per a day for these activities. Activities included (1) social networking/media (Instagram, twitter, Facebook) and chatting online (Facebook messenger, WhatsApp, Skype, Snapchat), (2) online gambling, (3) online gaming, (4) online shopping, (5) television (on-demand entertainment such as Netflix), (6) blogging, (7) video watching (e.g. YouTube), (8) web browsing, and (9) reading online blogs, news, articles, forum, and books.

#### Young’s internet addiction test (IAT)

The IAT was developed to determine the presence and severity of internet addiction in adulthood and is consisted of 20 questions ranked from “0 = not applicable” to “5 = always” (e.g., How often do you find that you stay online longer than you intended? ) [42]. It has six dimensions: salience (item 10, 12, 13, 15, and 19), excessive use (item 1, 2, 14, 18, and 20), neglect work (item 6, 8, and 9), anticipation (item 7 and 11), lack of control (item 5, 16, and 17), and neglect social life (item 3 and 4) [43]. It can be classified as four categories: normal amount of internet usage (scores between 0 and 30), mild internet addiction (scores between 31 and 49), moderate internet addiction (scores between 50 and 79), and severe internet addiction (scores between 80 and 100). Its reliability was determined as a = 0.889.

#### Adult ADHD Self-Report scale symptom (ASRS-v1.1) checklist

The ASRS-v1.1is used to evaluate symptom burden and identify the symptom profile. It consists of 18 items (6 items in Part A for identifying adults at-risk for, and 12 items in Part B to further assess the frequency of ADHD symptoms) ranked from “0 = never” to “4 = very often” (e.g., How often do you have problems remembering appointments or obligations? ). It compromises inattention (items 1–4, 7–11) and hyperactivity/impulsivity symptoms (items 5–6, 12–18) [44]. Severe symptoms of ADHD are indicated by high scores on this scale. Its reliability was determined as a = 0.907.

### Statistical analysis

JASP 0.16.2.0 and IBM SPSS Statistics 28 programs were used for data analysis. To estimate normality, the Kolmogorov-Smirnov test and Boxplots were used [45].

Pearson correlation was used to estimate the relationship among ADHD symptoms, internet addiction test with its subscales, the Stroop interference effect (response time (RT) to incongruent stimuli – RT to neutral condition), and the Stroop facilitation effect (RT to neutral condition - RT to congruent stimuli).

To estimate the differences in online activities between low and high ADHD symptoms, between low and high internet addiction symptoms, and between low and high inhibitory control deficits, participants were divided into the “Yes” group (those who reported an activity as among their top 5 most frequent activities) and the “No” group (those who did not reported an activity as being among their top 5 most frequent activities), for each internet activity. Then, three different t-tests were run to determine the differences between ADHD symptoms and online activities, between internet addiction and online activities, and between inhibitory control ability and online activities. To estimate the associations between a series of online activities and low/high inattention symptom, between a series of online activities and low/high internet addiction, and between a series of online activities and low/high inhibitory control deficits, the “Yes” groups and the “No” groups for each online activity were assigned as a multiple response sets. Then, Chi-square for multiple response sets was performed.

Moderation analysis using PROCESS Version 3.0 [46] was performed to examine a potential moderating role of inhibitory control in the relationship between inattention and internet addiction. Also, bootstrapping was used to find 95% confidence intervals around the 1000 resampling approach.

## Results

### The relationship among ADHD symptoms, internet addiction with its subscales, and the Stroop effects

A Pearson’s Correlation was conducted to determine the relationships among variables. The analysis revealed a moderately strong, positive correlation between inattention and internet addiction (*r (203)* = 0.560, *R*^*2*^ = 0.31, *p <*.001), hyperactivity/impulsivity and internet addiction (*r (203)* = 0.421, *R*^*2*^ = 0.18, *p <*.001), and between ASRS scores and internet addiction (*r (203)* = 0.533, *R*^*2*^ = 0.28, *p <*.001). See Table 1 for correlations between all self-report variables.

**Table 1:** The relationship among ADHD symptoms, internet addiction, and the Stroop effects

Variable				1		2		3	4		5		6				
1. Inattention		Pearson’s r		—													
		p-value		—													
2. Hyperactivity/impulsivity		Pearson’s r		0.701		—											
		p-value		< 0.001		—											
3. ASRS		Pearson’s r		0.924		0.920		—									
		p-value		< 0.001		< 0.001		—									
4. Internet addiction		Pearson’s r		0.560		0.421		0.533		—							
		p-value		< 0.001		< 0.001		< 0.001		—							
5. Stroop interference effect		Pearson’s r		0.054		0.032		0.046		0.011		—					
		p-value		0.446		0.653		0.510		0.871		—					
6. Stroop facilitation effect		Pearson’s r		-0.068		-0.050		-0.064		-0.018		-0.208			—		
		p-value		0.333		0.476		0.361		0.796		0.003			—		

### ADHD symptoms, internet addiction, and online activities

Levene’s Test for Equality of Variances was used to determine homogeneity of variance and it was found that there was homogeneity of variance. A t-test was performed for differences between ADHD symptom scores (continuous, dependent variable) and internet addiction (grouping variable: low vs. high internet addiction groups). It was found that the inattention symptom score in the high internet addiction group (15.22 ± 5.65) was significantly higher than in the low internet addiction group (9.26 ± 4.92) (*t*(203) = -8.043, *p* = < 0.001). Consequently, overall ADHD score in the high internet addiction group (27.82 ± 10.04) was significantly higher than the low internet addiction group (17.62 ± 9.66) (*t*(203) = -7.403, *p* = < 0.001).

A t-test was performed for differences between scores on the ADHD symptom scale for the different internet activities (grouping variable: “yes” and “no” groups for each activity). The results showed there were no group differences for ADHD symptoms (*p* >.05). Similarly, a t-test was performed for differences between internet addiction and internet activities (grouping variable: “yes” and “no” groups for each activity). The results again showed that there were no group differences in terms of the types of online activities participants mostly engaged in (*p* >.05). A t-test was performed for differences between scores on the inhibitory control ability for the different internet activities (grouping variable: “yes” and “no” groups for each activity). The results showed there were no group differences for inhibitory control ability (*p* >.05).

A separate Chi-square for multiple response sets was performed to estimate the associations between a series of online activities and low/high inattention symptoms. There were no differences between low and high inattention symptom group (*p* >.05) for online activities. The most commonly reported specific types of online activities by the high inattention group were video watching, social networking, web browsing, reading online blogs, and TV (See Table 2). A separate Chi-square for multiple response sets was performed to estimate the associations between a series of online activities and low/high internet addiction. There were no differences between low and high internet addiction group (*p* >.05) for online activities. The most commonly reported specific types of online activities by the high internet addiction group were social networking, video watching, web browsing, TV, and reading online blogs. A separate Chi-square for multiple response sets was performed to estimate the associations between a series of online activities and low/high inhibitory control deficits. There were no differences between low and high inhibitory control deficits group. The most commonly reported specific types of online activities by the high inhibitory control deficits group were video watching, social networking, web browsing, TV, and reading online blogs (See Table 2).

**Table 2:** Online activities in low/high inattention symptom, low/high internet addiction groups, and low/high inhibitory deficits groups. For all comparisons, *p* >.5

	Low Inattention Group (*n* = 112)	High Inattention Group (*n* = 93)
**Online Activities (%)**		
Social Networking	102 (91.1%)	84 (90.3%)
Online Gambling	10 (8.9%)	6 (6.5%)
Online Gaming	46 (41.1%)	38 (40.9%)
Online Shopping	53 (47.3%)	45 (48.4%)
TV	86 (76.8%)	54 (58.1%)
Blogging	3 (2.7%)	2 (2.2%)
Video Watching	97 (86.6%)	87 (93.5%)
Web Browsing	88 (78.6%)	76 (81.7%)
Reading Online Blogs	65 (58.0%)	63 (67.7%)
	**Low Internet Addiction Group (** ***n*** ** = 101)**	**High Internet Addiction Group (** ***n*** ** = 104)**
**Online Activities (%)**		
Social Networking	88 (87.1%)	98 (94.2%)
Online Gambling	8 (7.9%)	8 (7.7%)
Online Gaming	41(40.6%)	43 (41.3%)
Online Shopping	50 (49.5%)	48 (46.2%)
TV	74 (73.3%)	66 (63.5%)
Blogging	0 (0.0%)	5 (4.8%)
Video Watching	91 (90.1%)	93 (89.4%)
Web Browsing	81(80.2%)	83(79.8%)
Reading Online Blogs	65(64.4%)	63(60.6%)
	**Low Inhibitory Control Deficits Group (** ***n*** ** = 108)**	**High Inhibitory Control Deficits Group (** ***n*** ** = 97)**
**Online Activities (%)**		
Social Networking	100 (92.6%)	86 (88.72%)
Online Gambling	7 (6.5%)	9 (9.3%)
Online Gaming	42(38.9%)	42 (43.3%)
Online Shopping	51 (47.2%)	47 (48.5%)
TV	73 (67.6%)	67 (69.1%)
Blogging	2 (1.9%)	3 (3.1%)
Video Watching	97 (89.8%)	87 (89.7%)
Web Browsing	89(82.4%)	75(77.3%)
Reading Online Blogs	69(63.9%)	59(60.8%)

### Moderation analysis

Moderation analysis using PROCESS Version 3.0 (Hayes, 2013) was performed to examine a potential moderating role of inhibitory control in the relationship between inattention and internet addiction, and between inattention and internet addiction subscales.

The model explained 33% of the variation [*F*(3, 201) = 32.64, *p* < .01] and revealed inattention predicted an increase in IA for those with low (β low = .64, *p* < .01), moderate (β moderate = .52, *p* < .01) and high interference inhibition (β high = .40, *p* < .01) with the relationship getting weaker as inhibition deficit (Stroop interference) increased. HI scores were not a significant predictor (*p* = .43). Therefore, when controlling for HI, inhibitory control scores moderated the relationship between inattention and IA (see Table 3). The results extend those of the previous [33] by showing that low inhibitory scores also moderate the relationship between inattention and internet addiction. However, in the present data, the relationship between inattention and internet addiction gets weaker as inhibition deficit gets bigger.

**Table 3:** The moderation effect of inhibitory control when ADHD traits are predicting internet addiction

Effect	β	SE	95% Confidence Interval		*p*
			Lower Bound	Upper Bound	
Intercept	.006	.058	−.108	.120	.913
Inattention	.522	.081	.362	.683	< .01
Inhibitory control	−.029	.058	−.144	.085	.614
Inattention*Inhibitory control	−.119	.059	−.236	−.003	.045
Hyperactivity/impulsivity	.064	.081	−.097	.224	.434

Note: The coefficient here represents the standardized beta

## Conclusions

In this study, we aimed to further investigate the relationship between inattention and internet addiction whilst considering the effect of specific online activities. A further aim of the study was to replicate our previous finding showing inhibitory control ability moderated the relationship between internet addiction and inattention [37], and to see if inhibitory control ability was related to the type of online activity participants engage in. The findings showing a unique relationship between inattention and internet addiction [35, 37], and inhibition once again modulated the effect [37] but not that the previous finding was replicated. However, in contrast to the previous study [37] the present analysis revealed that the relationship between inattention and internet addiction actually weakened as inhibitory performance worsened indicating that poorer inhibition ability somehow protects against developing internet addiction. The reason for this difference is unclear and is discussed further below. Nevertheless, the most important contribution of the present work is that it shows that for the specific online activities, there were no group differences for high vs. low ADHD symptoms, high vs. low internet addiction, and high vs. low inhibitory control ability and that blogging activity was a predictor of internet addiction when other online activities were accounted for.

### Correlations among ADHD symptoms and internet addiction

The present study found that both inattention and hyperactivity/impulsivity were correlated with internet addiction and therefore aligned with previous research [33, 35, 47]. A recent meta-analysis concluded that while inattention, hyperactivity, and impulsivity were related to internet addiction, adults with inattention showed a stronger relationship compared to children or adolescents [48]. This study differs from the previous study in terms of the number of participants and the gender distribution of the participants. In this study, compared to our previous studies [35, 37], more participants were recruited. Also, compared to our previous studies, most of the participants were male (61%). It has been found that the correlation between inattention and internet addiction was more significant among female college students than among male college students [49], and men with ADHD have higher rates of hyperactive symptoms than women [50]. However, in our study, the correlation between inattention and internet addiction was not significant based on gender (*p* >.05), and females have higher rates of both inattention and hyperactivity/impulsivity symptoms compared to males. Therefore, we may have found that both symptoms are related to internet addiction.

### ADHD symptoms, internet addiction, inhibitory control ability, and online activities 1

In this study, we found no association between different online activities, each core ADHD symptom and the total ADHD symptom based on the ASRS-v1.1. In contrast to this result, Zhang et al., (2022) found that each core ADHD symptoms and the total ADHD symptom have different relationships with different online activities [38]. For example, while inattention, hyperactivity/impulsivity, and the total ADHD symptom score had a negative association with information download and online learning, they did not have any association with social communication. Furthermore, whereas inattention had a positive association with online gaming and no association with online shopping, the hyperactivity/impulsivity symptoms had a positive association with both online gaming and online shopping. Also, the total ADHD symptom score had no association with online gaming, online shopping and social communication. Compared to our study, the study had nine times more participants and the most of them were female (63.6%). Moreover, younger participants (*M*age = 19.35) were also included in the study compared to our study (*M*_age_=30.62). Panagiotidi & Overton (2018) [39], another study focusing on this association, divided ADHD scores to low and high ADHD groups, and they found results similar to the present study and showed that there were no group differences in online activities between low and ADHD groups for social networking, online shopping, and blogging, and group differences for online gaming, video game watching, MMORPGs, online gambling, and television. Compared our study, they recruited more participants (*n* = 420) and also their high internet addiction group consisted of adults whose internet addiction score were over 50 (*n* = 53). In addition, their most of their participants were female (55%) and their participants had higher inattention scores (*M* = 17.8) and hyperactivity/impulsivity scores (*M* = 14.2) than our participants (*M*_inattention_ = 12.28, *M*_hyperactivity/impulsivity_ = 10.51). Further research is needed to explore the possibility that online activity does differ between high and low levels of ADHD and internet activity.

Following these previous studies, to investigate the group differences in online activities based on internet addiction severity, we divided our sample into two groups: low and high internet addiction groups. We did not find group differences in online activities. Other researchers, Panagiotidi & Overton (2018) divided participants into two groups, internet addicted group and non-internet addicted group [39], and in alignment with our result, they did not find any differences for social networking, online shopping, online gambling, blogging, and online gaming and group differences in video surfing, video game watching, MMORPGs, and Television. However, Zhang et al., (2022) found that there were group differences between an internet addicted group and a non-internet addicted group [38]. Whereas the internet addicted group had a higher prevalence of online gaming, they had a lower prevalence of online learning compared to non-internet addicted group. In addition, they found that the internet addicted group reported more inattention and hyperactivity/impulsivity symptoms compared to non-internet addicted group. Our results are aligned with these results in that high internet addiction group reported more inattention and hyperactivity/impulsivity symptoms compared to the low internet addiction group.

In a novel contribution, the present study also focused on the differences between online activities and inhibitory control ability. We divided our participants into low and high inhibitory control deficit groups and found that online activities were not different between low and high inhibitory control deficits groups.

### Moderation analysis for the relationship between inattention and internet addiction

In our previous research, we concluded that although inhibitory control did not have a direct relationship with either inattention or internet addiction, inhibitory control moderated the relationship between inattention and internet addiction only in adults who experience high and moderate inhibitory control deficits [37]. Aligned with the findings, we found that even though inhibitory control did not have a direct relationship with either inattention or internet addiction, inhibitory control once again moderated the relationship between inattention and internet addiction in this study. However, in contrast to our previous study showing that inhibitory control moderated the relationship in adults at moderate, and high inhibitory control deficits, inhibitory control moderated the relationship in adults at all levels of inhibitory control ability in the present study but in the opposite direction to that reported by Aydin et al. (2024). That is, the relationship between inattention and internet addiction actually strengthened as inhibitory performance improved indicating that poorer inhibition ability somehow protects against developing internet addiction in those with inattention. The reason for this difference in the two studies is unclear. The Stroop effects were similar in the two studies: *M* = 55.35, *SD* = 44.12 in the previous study, and *M* = 44.35, *SD* = 40.58 in this study. The methods employed in the two studies were almost identical other than the inclusion of questions related to online activities in the present paper and a slight and seemingly unimportant difference in the Stroop task methods (the relative proportion of incongruent, neutral and congruent trials was equal in the present study but there was a smaller proportion of congruent trials in the previous study [37]. Both Stroop methods permit the measurement of interference (Incongruent – Neutral). Nevertheless, it is clear from the results of the two studies that that inhibitory control ability has a role to play in this relationship but future research will have to investigate the nature of the moderation to determine the role of inhibitory control ability.

In another study, researchers focused on motivational and executive dysfunctions in the association between ADHD and internet addiction, and they found that motivational dysfunction is a better predictor of internet addiction [32]. The difference between this study and our studies may derive from a different methodological approach. For example, while they focused on the relationship between ADHD and internet addiction, we focused on the relationship between inattention and internet addiction. They also divided participants to three groups: an executive dysfunction group, a motivational dysfunction group and a combined dysfunction group, based on their time 1 performances on cognitive tasks, including continuous performance test, stop-signal task, n-back task, delay discounting task, and the balloon analogue risk task. After six months (Time 2), they again measured their ADHD symptoms and internet addiction in these groups defined based on performance in time 1. Although there are methodological differences between these studies, they revealed that executive function or motivational function deficits may play a role in the relationship between ADHD symptoms, especially the inattention symptom, and internet addiction. Thus, future research could further explore the role of motivational dysfunction in the relationship between ADHD/inattention and internet addiction.

### The limitation of the study and the implications for future research

In the study, we recruited participants who are diagnosed neither ADHD nor internet addiction, and this can be accepted one of the limitations of the study. It has been shown that people who are diagnosed with ADHD [52] and diagnosed with internet addiction [32] have executive function deficits compared to healthy controls. However, in neither case is the evidence strong. Indeed, it is unclear the role executive function deficits play in ADHD, not every individual diagnosed with the condition may have a deficit [53]. Nevertheless, our studies show that the relationship between inattention and internet addiction increases as executive function impairment increases indicating that ADHD symptoms and executive functions interact in a way that leads to real-life consequences. Furthermore, another limitation is using questionnaires based on self-rating which can lead to self-report bias [54]. Therefore, it is recommended to replicate the study with adult who have been diagnosed with ADHD and internet addiction.

Another limitation of the study is focusing only on deficits in inhibitory control. It has been shown that both people with ADHD and with internet addiction showed deficits in many different cognitive domains such as working memory [32, 55], shifting [56, 57], and decision-making [51, 58]. Therefore, it is recommended that future studies measure these other cognitive functions and investigates their role in the relationship between inattention and internet addiction.

Despite its limitations, this study is the first study to examine the relationship between ADHD symptoms, internet addiction, online activities and inhibitory control in the general population. As found previously [37], inhibitory control impairments moderate the relationship between inattention and internet addiction. These results can contribute to understanding of the relationship between internet addiction and ADHD in clinical practice, development of new efficient treatment strategies, and determination of precautions for internet addiction.

To sum up, this study is the first study to explore how specific online activities might modify the relationship between inattention, internet addiction and inhibitory control. Consistent with previous research we showed that inattention predicted internet addiction, and that this relationship is moderated by inhibitory control ability in that the relationship between inattention and internet addiction increases as executive function impairment increases. These results indicate that ADHD symptoms and executive function impairments interact in a way that leads to real-life consequences, even when there is no relationship between those symptoms and executive function performance. Furthermore, we showed that no single online activity is responsible for the relationship between inattention and internet addiction, nor does inhibitory control ability determine which online activity participants engage in.

## Data Availability

The data are available from the corresponding author on reasonable request.

## Footnotes

1 Panagioti and Overton created their high and low IA groups by splitting their cohort into those that scored >50 (Highs) and those scoring <50 (lows). This gave them 33 highs and 367 lows. Using the same approach in our dataset gave us 9 highs and 196 lows. When we run the analysis using the grouping method employed by Panagioti and Overton, the outcome did not change, there were no differences between highs and lows in any of the chosen online activities. To further check for differences, we change the grouping cut-off to 40 which gave us 17 highs (roughly 10% of the sample - akin to Panagioti & Overton) and still the results were not significant.
